# Consistent changes in global gene expression patterns despite strong variation in individual gene expression in the male mouse hippocampus following early life stress

**DOI:** 10.1016/j.ynstr.2026.100808

**Published:** 2026-03-30

**Authors:** Jeniffer Sanguino-Gómez, Jacobus C. Buurstede, Eduardo H.L. Umeoka, Marcia Santos da Silva Umeoka, Max Gentenaar, Norberto Garcia-Cairasco, Mario F. Juruena, Wilbert P. Vermeij, Jan H.J. Hoeijmakers, Harm J. Krugers, Marian Joëls, Onno C. Meijer

**Affiliations:** a Department of Medicine, Division of Endocrinology, Leiden University Medical Center, Leiden, the Netherlands; b Brain Plasticity Group, SILS-CNS, University of Amsterdam, Amsterdam, the Netherlands; c Ribeirão Preto School of Medicine, University of São Paulo, Ribeirão Preto, SP, Brazil; d Department of Psychological Medicine, Institute of Psychiatry, Psychology and Neurosciences-King's College London, UK; e Princess Máxima Center for Pediatric Oncology, Utrecht, the Netherlands; f Oncode Institute, Utrecht, the Netherlands; g Department of Molecular Genetics, Erasmus MC Cancer Institute, Erasmus University Medical Center Rotterdam, Rotterdam, the Netherlands; h Institute for Genome Stability in Ageing and Disease, Cologne Excellence Cluster for Cellular Stress Responses in Aging-Associated Diseases (CECAD), University of Cologne, Cologne, Germany; i University Medical Center Groningen, University of Groningen, Groningen, the Netherlands

## Abstract

Exposure to excessive and/or chronic stressors during early life is a well-established risk factor for later life stress-related mood disorders. Glucocorticoids are often implied as mediators of the long-term effects of early life stress (ELS), given their powerful transcriptional effects via the glucocorticoid receptor (GR). Previous work in rodents showed that the GR-antagonist RU486 administered during early puberty can reverse some of the behavioral and cellular effects of ELS. Here, we investigated the long-term transcriptional effects of ELS, using the limited bedding and nesting model, on the hippocampus of adult or adolescent male mice. The effects on the adult dorsal hippocampal transcriptome, established in two separate cohorts (of two labs), were found to be inconsistent. The same inconsistency was observed when investigating the transcriptome in adolescent mice – thus examining the tissue with a shorter interval after ELS – in tissue from two cohorts where virtually all sources of variation were controlled for, involving the same ELS protocol, laboratory, and experimenter. Interestingly, despite the limited overlap of individual differentially expressed genes between cohorts, we did observe a consistent pattern at a more global transcriptional level: a preferential upregulation of shorter genes and progressive downregulation with increasing gene length (as a continuous variable) after ELS, consistent with a gene-length-dependent transcription decline (GLTD) pattern. This GLTD pattern was accompanied by an enrichment of aging-associated transcriptomic changes in glial and vascular brain cell types during adolescence, a signature that was attenuated in adulthood. Together, these findings indicate that while individual gene-level responses to ELS are highly variable, robust and reproducible transcriptional patterns emerge at the level of global gene architecture (based on length) and cellular aging signatures.

## 1 Introduction

When faced with an acute stressor the body's first response is to appropriately deal with the current threat, after which homeostasis has to be reinstated (Chrousos, 2009; Tsigos et al., 2000). This involves activation of the hypothalamic-pituitary-adrenal (HPA) axis, resulting in the secretion of predominantly cortisol in humans and corticosterone in most rodent species. These steroid hormones not only regulate the later phases of the stress response that help restoration of homeostasis but also support processes that help adaptation to future stressors (Herman et al., 2016). When environmental challenges occur during critical periods of life (such as the perinatal period or adolescence), the consequences can persist for a long time, as demonstrated already in the 1950s by the seminal work of Seymour (Gig) Levine, who stated that early experiences can modify later reactivity of the brain under stress conditions (Levine, 1957, 2005). Adaptations to perinatal stress may lead to long-term changes in homeostatic setpoints, a phenomenon called allostasis (McEwen and Wingfield, 2003). This adaptive capacity in general likely contributes to resilience (the capacity to maintain health despite potential stressors (Hermans et al., 2025)) when the resulting adaptations are matched to the individual's future environment. However, when this match fails, the same stress-induced adaptations may increase vulnerability and promote maladaptive cognitive, emotional and physiological outcomes (Nederhof and Schmidt, 2012).

These processes are dynamic and particularly relevant upon early life stress (ELS; mice) or childhood trauma (humans), which may involve either multiple acute stressors or a single very strong stressor (Birnie and Baram, 2025). The adaptive changes in these cases can create a context poised to deal with similar challenges during adulthood, resulting in a brain that is “programmed” to deal with a life filled with a series of adversities (Champagne et al., 2008; Chen and Baram, 2016; de Kloet et al., 2005; Santarelli et al., 2017; Godoy et al., 2018). This programming may involve the high endogenous glucocorticoid levels resulting from the stressor(s), activating glucocorticoid receptors (GR) expressed throughout the brain (Gans and Coffman, 2021). While these programming effects might be beneficial for dealing with future stressors, they may also negatively interfere with cognitive and emotional processes in “safe” situations, and in this way form a mismatch with later life circumstances (Daskalakis et al., 2013; Gluckman et al., 2007; Schmidt, 2011). In fact, childhood trauma correlates with increased incidence, severity, and treatment resistance across psychiatric diagnoses (Kuzminskaite et al., 2022; Stanton et al., 2020).

Various rodent models (mostly in mice) have been developed over the past decades to study the mechanism(s) underlying the effects of ELS, including maternal separation and the limited bedding and nesting (LBN) paradigm (Millstein and Holmes, 2007; Rice et al., 2008). The latter is considered a translatable model in which the absence of sufficient nesting materials negatively affects the predictability of maternal care, an important developmental factor (Meaney, 2001; Orso et al., 2019). The LBN paradigm is characterized by lower body weight at weaning, and changes in multiple behavioral domains (e.g. anxiety, learning, and social) in later life, particularly in male rodents (Bonapersona et al., 2019; Walker et al., 2017). Learning deficits are robust, for instance in the fear conditioning task, where adult male mice exposed to ELS show impaired fear acquisition (Sanguino-Gómez and Krugers, 2024; Sanguino-Gómez et al., 2024), impaired memory retrieval for both contextual and auditory cues (Sanguino-Gómez and Krugers, 2024; Lesuis et al., 2019), and an inability to discriminate between safe (cue-off) and non-safe (cue-on) periods (Arp et al., 2016). Remarkably, across three studies, RU486 administration during early puberty normalized distinct fear-related deficits in ELS-exposed males: impaired discrimination between safe and non-safe cues (Arp et al., 2016), impaired fear memory consolidation (Loi et al., 2017) and impaired fear acquisition (Sanguino-Gómez and Krugers, 2024). This demonstrated for the first time that curative interventions after ELS might be an attainable goal. Through which mechanisms ELS affects behavior in adulthood, and how this is (in part) normalized by a brief intervention in adolescence, is unclear. However, given that GR is a transcription factor, and given the efficacy of the GR antagonists, the underlying mechanism may be found at the level of transcription or the epigenetic regulation thereof.

In this study, we set out to investigate how ELS long-lastingly affects the male mouse hippocampus transcriptome. To this end, we used multiple cohorts of ELS and control mice, with or without adolescent GR antagonist intervention, and assessed the corresponding transcriptome changes. By investigating the effect of ELS in multiple cohorts of mice, we tried to assess the reproducibility and replicability of the effects at the transcriptional level.

## 2 Materials and methods

### 2.1 Animals

Four cohorts of animals were used in the study, which was performed in two laboratories, following the same experimental setup. C57BL6 mice were bred in-house (originally acquired from Envigo, the Netherlands, and Campinas-SP, Brazil). Animals had access to *ad libitum* food and water and a light/dark cycle of 12 h/12 h (light period starting at 8:00 a.m.). Litters consisted of a maximum of six pups, with at least one female randomly assigned to either the ELS or control conditions. At PND2, ELS was imposed using the limited bedding and nesting material (LBN) paradigm (Arp et al., 2016; Rice et al., 2008). After PND9, the litters were transferred to standard cages, and pups were kept with the dams until weaning (PND21), after which the male animals were group-housed (up to five animals per cage) and left undisturbed (apart from treatment) until sacrifice at PND31 (two adolescent reproducibility cohorts) or PND120 (two adult cohorts). In this study, we focused on male mice, given the rather weak behavioral effects earlier seen at a meta-level in females (Bonapersona et al., 2021). Hippocampal tissue was directly dissected after sacrifice in the morning to minimize the impact of circadian-induced fluctuations in corticosterone levels on molecular changes (den Boon et al., 2019) and snap-frozen for molecular analysis. All animal experiments were approved by the national Animal Ethics Committees (University of Amsterdam and Ribeirão Preto School of Medicine of the University of São Paulo) and carried out at the University of Amsterdam or the University of São Paulo under the approval of the local Animal Welfare Body under the protocol number 111/2018 in São Paulo and in compliance with the EU directive 2010/63/EU in Amsterdam.

The cohorts were structured as follows: the two adult cohorts (São Paulo and Amsterdam; N = 24 each) consisted of four experimental groups: Control-Vehicle, Control-RU486, ELS-Vehicle, and ELS-RU486 (n = 6 per group). The two adolescent cohorts consisted of two experimental groups each: Control-Vehicle and ELS-Vehicle (cohort 1: N = 8, n = 4 per group; cohort 2: N = 12, n = 6 per group).

### 2.2 Limited bedding and nesting paradigm

From PND2 to PND9, control dams and pups were placed in cages with a standard amount of bedding material (a 2 cm thick layer of sawdust (approximately 100g) and a square of cotton nesting material (5 cm by 5 cm). ELS animals were provided with one-third the standard amount of bedding and nesting material compared to controls. Additionally, a stainless-steel grid (22 cm × 16.5 cm with a mesh size of 5 mm × 5 mm, in-house made) was placed 1 cm above the cage floor to impede the use of sawdust for nesting. Mice from both groups remained undisturbed until PND 9, when dams and pups were returned to standard housing conditions. Animals remained undisturbed until PND 21 when the mice were weaned. The body weight of the pups was assessed before (PND2) and directly after ELS (PND9) or control treatment, and during treatment to confirm the effectiveness of the LBN paradigm.

### 2.3 Conditions and treatment

In the adult cohorts (PND120), animals which underwent ELS or control rearing were treated three times with GR antagonist RU486 (Mifepristone; 10 mg/kg, Sigma) or vehicle (0.25% carboxymethylcellulose, 0.2% tween and 0.9% NaCl in water) during adolescence (PND28-30) by intraperitoneal injection, following an earlier paradigm in rodents (Arp et al., 2016; Loi et al., 2017; Sanguino-Gómez and Krugers, 2024). Animals were left under standard housing conditions until sacrifice at PND120. Cohort 1 was first performed at the University of São Paulo in Ribeirão Preto (Adult cohort 1) and repeated at the University of Amsterdam (Adult cohort 2). In the adolescent cohorts (PND31), animals underwent ELS or control rearing, injected similarly and were killed at PND 31, the morning after the last day of treatment. Both adolescent cohorts were performed at the University of Amsterdam. In all cases, hippocampi were rapidly dissected from freshly extracted brains and quickly snap-frozen. Dissection procedures were kept comparable across all experimental settings.

### 2.4 RNA sequencing

Total RNA was isolated from dorsal hippocampal (i.e. the ventral-most 25% was excluded) tissue of adult ELS and control animals (PND120) homogenized in the lysis buffer of the NucleoSpin RNA kit (Macherey-Nagel). Total RNA was isolated according to the manufacturer's protocol and was sent for transcriptome sequencing at BGI Genomics (Beijing, China). Hippocampal tissue of ELS and control animals was sent to BGI Genomics for total RNA isolation using RNeasy kit (Qiagen) and transcriptome sequencing. All RNA samples were required to meet BGI's minimum quality criteria for library preparation: RNA Integrity Number (RIN) ≥ 7.0 and 28S/18S ratio ≥1.0. Following quality control, mRNA was enriched using poly(A) selection according to BGI standard protocols. Stranded mRNA libraries were constructed and sequenced on the DNBseq-G400 platform with 100 bp paired-end reads, generating >20 million reads per sample. Raw sequencing data were filtered using SOAPnuke (BGI) to remove adapter sequences, low-quality reads and reads with a high proportion of undetermined base calls (N). Post-filtering quality metrics confirmed high data quality across all samples (Q20 > 96%, GC content ∼50%). Post-alignment quality control was performed using MultiQC (Ewels et al., 2016).

### 2.5 RNA-seq data analysis

#### 2.5.1 Data processing

The RNA-seq pipeline (version 4.1.0), published as part of BioWDL, was used for read quality control, alignment and quantification. BioWDL contains the main sequencing analysis pipelines and workflows developed at Leiden University Medical Center by the sequencing analysis support core with code being accessible at https://biowdl.github.io/.

Quality control was performed using FastQC and MultiQC. Reads were aligned to *Mus musculus* genome version 10 (mm10) using STAR (version 2.7.3a). The gene-read quantification was performed using HTSeq-count (version 0.12.4) on Ensembl release 97 of mm10. HTSeq-count output files were merged into a count matrix per experiment as input for differential gene expression analysis.

#### 2.5.2 Differential expression analysis

DEseq2 (version 1.29.4) was used for normalization of the count data (median of ratios method) and identification of differentially expressed genes (Love et al., 2014). For the differential expression analysis, all genes which were expressed in a minimum of four (for the PND120 cohorts) or three (for the PND31 cohorts) replicates with >20 normalized counts for at least one of the groups were selected. This threshold is based on the minimum group size in each cohort (4-6 animals per group in the adolescent cohorts versus 6 animals per group in the adult cohorts), ensuring genes expressed in at least half of the smallest groups are retained for analysis. One sample of PND120 cohort 1 was identified as an outlier (sample 21 of the ELS RU486 group) based on principal component analysis (Supplementary Fig. 1A–D), showing substantial deviation from other samples; this sample was excluded from subsequent analysis. Pairwise comparisons of groups within experiments were analyzed, and a false discovery rate-adjusted p-value of 0.05 was used as a cut-off for detecting differential gene expression (Supplementary Table 1).

#### 2.5.3 Gene Ontology analysis

Gene ontology (GO) enrichment analysis was performed on all genes differentially expressed after ELS of PND120 cohort 1 with the ViSEAGO package (version 1.4.0), using Fisher's exact test with 0.01 as a significance cut-off (Brionne et al., 2019) (Supplementary Table 2).

#### 2.5.4 Differential exon usage analysis

Differential exon usage analysis was performed on all vehicle ELS and control animals with the DEXSeq package (1.36.0) (Anders et al., 2012) with the default settings and a false discovery rate adjusted p-value of 0.01 for detection of differential exon usage (Supplementary Table 3).

#### 2.5.5 Gene length-dependent transcriptional analysis

Gene length-dependent analysis was performed following the approach of Vermeij et al. (2016) and van Galen et al. (2025). Gene length information was retrieved from the Ensembl database (release 97) using the biomaRt R/Bioconductor package (version 2.46.3) (Durinck et al., 2009).

All expressed genes with TMM (Trimmed Mean of M-values) normalization >1 (>10,000 genes per cohort) were selected and ordered by gene length ensuring that the bin containing the longest genes contained exactly 500 genes. The incomplete shortest bin was excluded from analysis. TMM normalization values were extracted from the DESeq2 normalization output. Genes were grouped into consecutive bins of 500 genes each based on their genomic length. For each bin, the percentage of upregulated genes was calculated as the ratio of upregulated to total differentially expressed genes within that bin (Supplementary Table 4), and plotted against the mean gene length of that bin.

#### 2.5.6 Gene-set enrichment analysis

Gene-set enrichment analysis (GSEA) was performed on all expressed genes using the clusterProfiler package (version 4.10.0) (Wu et al., 2021), which implements the fgsea algorithm (Korotkevich et al., 2016). Cell type-specific aging signature gene sets were obtained from the Tabula Muris Senis (TMS) (The Tabula Muris Consortium, 2020). To ensure biological relevance to hippocampal tissue, only brain-derived TMS gene sets were included in the analysis (9 cell types: astrocytes, endothelial cells, interneurons, macrophages, microglia, neurons, oligodendrocytes, oligodendrocyte precursor cells, and pericytes), while aging signatures derived from peripheral tissues were excluded. Genes were ranked by a π-value, calculated by multiplying the log2(fold change) by −log10(adjusted p-value) to retain both significance and directionality of change (Xiao et al., 2014) (Supplementary Table 5). GSEA was run with 10,000 permutations, Benjamini-Hochberg correction for multiple testing, and minGSSize and maxGSSize set to 1 and 5,000, respectively. GSEA plots were generated using enrichplot (version 1.22.0).

### 2.6 Statistical analysis

Animals were randomly assigned to condition and treatment and data analysis was conducted by an experimenter who was blinded to the experimental condition. Sample size was determined a priori by conducting a power analysis with GPower 3.1. For RNA-seq analysis, we opted (when possible) to sequence 6 samples per group, which aligns with established recommendations for detecting differential gene expression with medium-to-large effect sizes in brain tissue (0.8 power to detect large effect sizes: log_2_FC ≥ 1.0, Cohen's d ≥ 1.2; reasonable power for medium-large effects: log_2_FC ∼0.7–0.8, d ∼0.8) (Schurch et al., 2016). We acknowledge potential power constraints for smaller effect sizes and proactively addressed them through a replication approach including separate and pooled analyses of cohorts at identical developmental stages to increase sample size. Statistical analysis and data visualization were performed using R version 4.5.2 and RStudio version 2025.9.2.418.

Data was screened by examining scatter plots for linearity, conducting Shapiro-Wilk tests for normality of residuals, and assessing homogeneity of variance using Levene's test. Residual plots were inspected to verify homoscedasticity and identify potential outliers.

Due to the nature of the experiment, the ELS variable is intrinsically bound to a specific litter. Therefore, body weights were analyzed, including litter as a random factor using a linear mixed model (Aarts et al., 2014; Yu et al., 2022) using the lme4 (v1.1.37) and lmerTest (v3.1.3) packages (Bates et al., 2015; Kuznetsova et al., 2017). To assess reproducibility of RU486-responsive signatures, Condition × Treatment interactions were tested for each cohort and cross-cohort reproducibility was evaluated using inverse-variance weighted and equal-weight meta-analyses with the package metafor (v4.4-0; Viechtbauer, 2010).

Gene length-dependent transcriptional responses were analyzed using linear regression with log_10_-transformed gene length as a continuous predictor. To test whether slopes differed between cohorts, we performed linear regression with an interaction term between gene length and cohort. Model comparisons were performed using ANOVA. Pearson correlations quantified the strength of the relationship between gene length and transcript ratio. Individual gene-level validation of the DEGs was performed using Mann-Whitney U tests. Separate analyses were performed for each developmental stage and cohort and models comparing cohorts within experiments were tested as well. Statistical significance was set at α = 0.05.

## 3 Results

### 3.1 Effect of ELS and RU486 intervention on genome-wide transcriptome of the adult male dorsal hippocampus

**Fig. 1:**
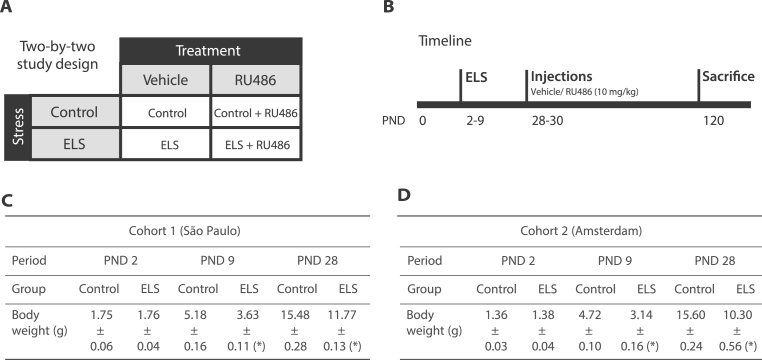
**Study design, experimental timeline and body weights. A**. Two-by-two design used to study the effects of early-life stress (ELS) and RU486. **B**. Experimental timeline of the experiment. Male mice were assigned to control or ELS conditions from postnatal day (PND) 2–9, with ELS induced using the limited bedding and nesting (LBN) paradigm. During adolescence (PND 28–30), mice received intraperitoneal injections of vehicle or RU486 (10 mg/kg). Animals were sacrificed at PND120. **C-D.** Body weights (in grams ± standard error of the mean - SEM) of experimental animals measured at PND 2 (prior to group assignment), PND 9 (immediately following control or ELS rearing conditions) and adolescence (average PND 28-30, when treatment was administered) in Adult cohort 1 (São Paulo) (C) and in an independent validation cohort (Adult cohort 2 (Amsterdam)) (D). **∗**: ELS effect. Effect p ≤ 0.05.

Earlier studies in male rodents observed a reversal of ELS-induced changes in behavior by intervention with the GR antagonist RU486 (Arp et al., 2016; Loi et al., 2017; Sanguino-Gómez and Krugers, 2024). To determine the long-lasting molecular effects of ELS and investigate the reported normalization by adolescent RU486 intervention, we used a two-by-two study design that followed previous work (Fig. 1A). ELS was imposed by the limited bedding and nesting (LBN) paradigm from PND 2-9, with control animals housed in standard conditions. After PND9, all animals were housed in standard conditions. RU486 or vehicle control was administered three times (at PND 28,29,30 - Arp et al., 2016; Loi et al., 2017; Sanguino-Gómez and Krugers, 2024). All animals were then left undisturbed and were sacrificed at PND120 (Fig. 1B). This experimental design was applied to two different cohorts and carried out by the same experimenter, though at different locations (São Paulo and Amsterdam). In both cases, before the onset of the LBN paradigm at PND2, the body weight of the (prospective) ELS and control mice of both cohorts was comparable (Adult cohort 1 (São Paulo): t(12) = 0.510, p = 0.619; Adult cohort 2 (Amsterdam): t(15) = 0.064, p = 0.950), whereas, at PND9, the ELS animals weighed less than their respective controls (Adult cohort 1 (São Paulo): t(12) = −4.073, p = 0.002; Adult cohort 2 (Amsterdam): t(15) = −4.639, p < 0.001) and this effect persisted till adolescence (Adult cohort 1 (São Paulo): t(12) = −5.782, p < 0.001; Adult cohort 2 (Amsterdam): t(15) = −3.074, p = 0.008) (Fig. 1C–D).

Firstly, as a control, we compared baseline transcriptional profiles of control animals between cohorts using correlations across all expressed genes. Control samples showed high between-cohort correlations (r = 0.950 ± 0.006), comparable to within-cohort correlations (Adult cohort 1 (São Paulo):r = 0.993 ± 0.001; Adult cohort 2 (Amsterdam): r = 0.991 ± 0.003). The 4.4% difference between average within-cohort/between-cohort correlation reflects expected technical variation (Leek et al., 2010). In fact, the between-cohort correlation indicates very high transcriptional similarity (90% shared variance), confirming that control animals maintained comparable baseline transcriptional profiles across experimental locations.

**Fig. 2:**
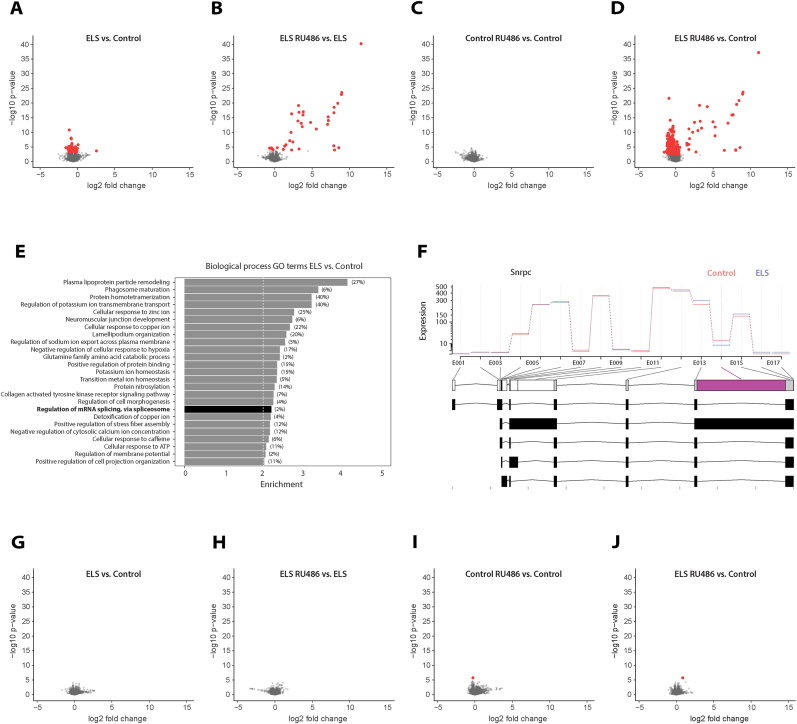
**Early life stress induces inconsistent transcriptional changes in the dorsal hippocampus at adult age (PND120). A–D.** Volcano plots showing differentially expressed genes (DEGs) for each experimental contrast in Adult cohort 1 (São Paulo). Red dots indicate DEGs at adjusted p-value <0.05. **E**. Gene Ontology (GO) enrichment analysis of biological processes for all DEGs in ELS versus control groups. Regulation of mRNA splicing is highlighted in bold. **F**. Representative example of differential exon usage showing significant changes in exon 14 of *Snrpc*, confirming altered mRNA splicing following ELS. **G–J**. Volcano plots showing DEGs for each contrast in an independent validation cohort (Adult cohort 2 (Amsterdam)). Red dots indicate DEGs at adjusted p-value <0.05. Adult cohort 1 (São Paulo): N_Control-Vehicle_ = 6 N_Control-RU486_ = 6 N_ELS-Vehicle_ = 6 N_ELS-RU486_ = 6. Adult cohort 2 (Amsterdam): N_Control-Vehicle_ = 6 N_Control-RU486_ = 6 N_ELS-Vehicle_ = 6 N_ELS-RU486_ = 6.

In Adult cohort 1 (São Paulo), differential expression analyses of the right hippocampus identified 116 differentially expressed genes (DEGs) after ELS (40 up- and 76 downregulated, Fig. 2A). To determine which processes were affected in adulthood after ELS, we performed GO-term enrichment analysis, which reported 25 biological processes including the regulation of mRNA splicing via the spliceosome (Fig. 2E). Differential exon usage analysis between ELS and control animals identified 57 differentially used exons (in 53 genes Supplementary Table 3, example gene: *Snrpc*, Fig. 2F), confirming that mRNA splicing was affected by ELS in this adult cohort. Intervention during adolescence with three times RU486 injection after ELS significantly changed the expression of 36 genes (31 up- and 5 downregulated) compared to vehicle-treated ELS animals (Fig. 2B), while the intervention with RU486 did not alter the transcriptome in control animals (Fig. 2C). The largest number of DEGs (715; 199 up- and 516 downregulated, Fig. 2D) was identified between RU486-injected ELS animals and vehicle-treated controls. The lack of reversal of ELS effects by RU486 intervention was confirmed by the minimal overlap of DEGs (one gene, *Trank1*) between the contrasts ELS-RU486 vs. ELS and ELS vs. Control.

We next aimed to replicate the DEGs that were identified in Adult cohort 1 (São Paulo). To address this, we performed an additional transcriptome analysis on the *left* dorsal hippocampus of a second cohort – Adult cohort 2 (Amsterdam)- generated by the same experimenter. (The right hippocampus from this cohort was used for chromatin analysis upon ELS (unpublished data)). In Cohort 2 (Amsterdam), we again validated the decrease in body weight at PND9 after ELS (Fig. 1D). However, in these animals, we found no effect of ELS on the transcriptome (Fig. 2G), nor did we observe any effect of the RU486 intervention compared to the vehicle-treated ELS group (Fig. 2H). Adolescent intervention with RU486 in control animals altered the expression of a single gene (Fig. 2I), and RU486-injected ELS animals compared to vehicle-treated controls also resulted in one DEG (Fig. 2J).

Side-by-side visualization of the ELS and RU486 intervention effects on the dorsal hippocampal transcriptome of both adult cohorts highlighted the inconsistency of the transcriptional effects. Formal analysis of RU486-mediated modulation of ELS effects (interaction effect) identified 49 genes with significant interactions in Adult cohort 1 (São Paulo):(48 upregulated, 1 downregulated; FDR <0.05), while Adult cohort 2 (Amsterdam) showed no significant interactions. Meta-analysis identified Aldob (aldolase B) as the only reproducibly RU486-responsive gene across cohorts, though with opposite directional effects (Cohort 1: log2FC = +2.59; Cohort 2: log2FC = −0.36). These data indicate that ELS can long-lastingly alter the dorsal hippocampal transcriptome, but did not do so in a consistent and reproducible manner in both cohorts, despite both cohorts undergoing early life stress strictly following the LBN paradigm performed by the same experimenter.

### 3.2 Effects of ELS on the adolescent genome-wide hippocampal transcriptome

The inconsistent results found at the transcriptome levels may be attributed to various factors, which all – to varying extent – will influence the eventual transcriptional signature obtained. Often discussed factors for ELS models are differences in the background of the animals, the researcher performing the LBN, and the laboratory in which the experiments were conducted (Walker et al., 2017). Moreover, our comparison relied on the assumption that the left and right dorsal hippocampal transcriptomes are comparable (see further: Discussion). We also considered that long-lasting molecular effects might be transient and fade out over time, introducing another factor that might contribute to the inconsistency we observed in adulthood (PND120).

**Fig. 3:**
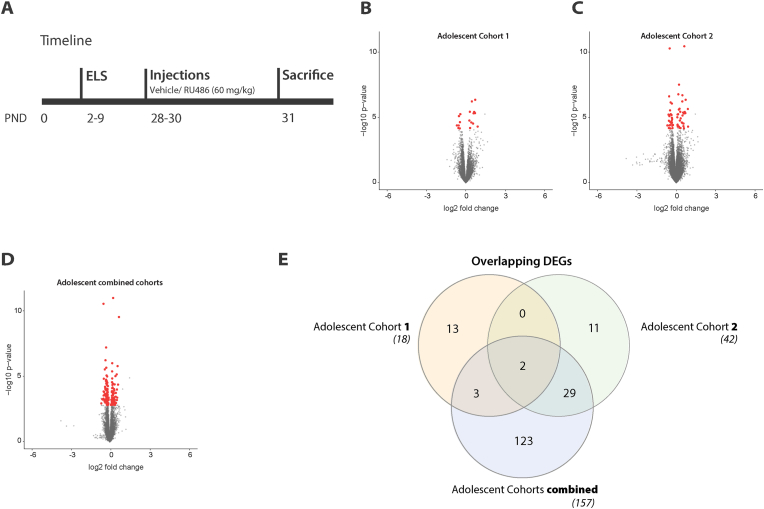
Early life stress induces inconsistent transcriptional changes in the whole hippocampus during adolescence (PND31). **A**. Experimental timeline of the experiment. Male mice were assigned to control or ELS conditions from postnatal day (PND) 2–9, with ELS induced using the limited bedding and nesting (LBN) paradigm. During adolescence (PND 28–30), mice received intraperitoneal injections of vehicle or RU486 (10 mg/kg) and were sacrificed one day after the last treatment (PND 31). **B-C**. Volcano plots showing differentially expressed genes (DEGs) between control and ELS animals in two independent cohorts. Red dots indicate DEGs at adjusted p-value <0.05. **D**. Volcano plots showing differentially expressed genes (DEGs) between control and ELS animals in two cohorts combined. Red dots indicate DEGs at adjusted p-value <0.05. **E**. Venn diagram displaying the number of overlapping DEGs across individual cohorts and the combined analysis. Adolescent cohort 1: N_Control-Vehicle_ = 4 N_ELS-Vehicle_ = 4. Adolescent cohort 2: N_Control-Vehicle_ = 6 N_ELS-Vehicle_ = 6.

To rule out the influence of all these factors, we performed another experiment to study the effects of ELS, and the consistency thereof, on the hippocampal transcriptome. We chose to investigate the transcriptomic changes after ELS during adolescence (at PND31, i.e. one day after the final RU486 treatment in the first two cohorts), hypothesizing that we would detect a stronger effect by ELS when examining the transcriptome shortly after the intervention period (10 days after weaning). A single researcher performed the two adolescent reproducibility cohorts back-to-back (within 3 months) with an identical experimental design in the same facility, keeping all variables as comparable as possible (Fig. 3A).

We first confirmed that the adolescent cohorts replicated the body weight phenotype observed in the adult cohorts. Before the onset of the LBN paradigm at PND2, the body weight of the (prospective) ELS and control mice was comparable in both cohorts (Adolescent cohort 1: t(10) = 0.604, p = 0.559; Adolescent cohort 2: t(15) = 1.406, p = 0.180). However, at PND9, ELS animals weighed significantly less than their respective controls (Cohort 1 t(10) = −5.456, p < 0.001; Cohort 2: t(15) = −3.439, p = 0.004) and this effect remained till the end of the experiment (Adolescent cohort 1: t(10) = −2.737, p = 0.021; Adolescent cohort 2: t(15) = −3.108, p = 0.007) (Supplementary Fig. 2A–B).

Before examining ELS effects, we verified that baseline transcriptional profiles were comparable between adolescent cohorts at PND31. Within-cohort correlations (Adolescent cohort 1: r = 0.995 ± 0.000; Adolescent cohort 2: r = 0.994 ± 0.001) were comparable to between-cohort correlations (r = 0.992 ± 0.002), with a 0.3% difference (average within-cohort/between-cohort) indicating excellent transcriptional similarity (98% shared variance) when the same experimenter, facility, and hemisphere were maintained. Despite these comparable baseline profiles, ELS-induced DEGs showed limited reproducibility between cohorts. Adolescent cohort 1 identified 18 DEGs (11 up-, 7 downregulated, Fig. 3B), while Adolescent cohort 2 identified 42 DEGs (24 up-, 18 downregulated, Fig. 3C). Only two genes (*Lct* and *Meg3*) were differentially expressed (downregulated and upregulated respectively) in both adolescent cohorts (Fig. 3E). This minimal overlap between highly comparable cohorts indicates that individual gene-level ELS responses are not reproducible, despite identical conditions and comparable baseline transcriptional profiles.

The adolescent reproducibility cohorts were performed in series, and the samples were processed simultaneously. Therefore, the groups could be pooled to increase the power of the analysis and assess the contribution of group size. Pooling increased the number of identified DEGs to 157 (66 up- and 91 downregulated, Fig. 3D), which showed that larger group sizes indeed aided the detection of DEGs. However, overlap-analysis of all DEGs revealed that the expression of 24 genes was no longer significantly altered after pooling both adolescent reproducibility cohorts (Fig. 3E). This may be an indication that ELS differently affected both cohorts - or part thereof - and that the inconsistent results were not merely driven by lack of power (Fig. 3E). This experiment showed that the effects of ELS on the adolescent hippocampal transcriptome were inconsistent even in tightly controlled conditions. With a bigger sample size, the number of significant differences increased, but the fold-change of these genes was very modest.

### 3.3 Effects of ELS on gene length-dependent transcriptional patterns and brain aging in the adolescent and adult hippocampus

Given the modest and inconsistent effects of ELS on gene expression between the two cohorts at PND31, we next investigated whether ELS might induce more global, systematic changes in gene expression patterns. Specifically, we examined whether ELS induces a gene-length-dependent transcriptional decline (GLTD): a pattern where longer genes show preferential downregulation relative to shorter genes. Previous studies have shown that transcription-blocking DNA damage disproportionately affects long genes, as they accumulate more stochastic lesions and become more transcriptionally impaired than short genes (Vermeij et al., 2016; Gyenis et al., 2023). Critically, GLTD can be induced by various types of stressors, including both physical stressors (nutrients, exercise, oxidative stress, radiation) and psychological stressors (Soheili-Nezhad et al., 2024), making it a potentially more robust and systematic ELS signature than the modest, inconsistent individual gene changes observed between cohorts.

**Fig. 4:**
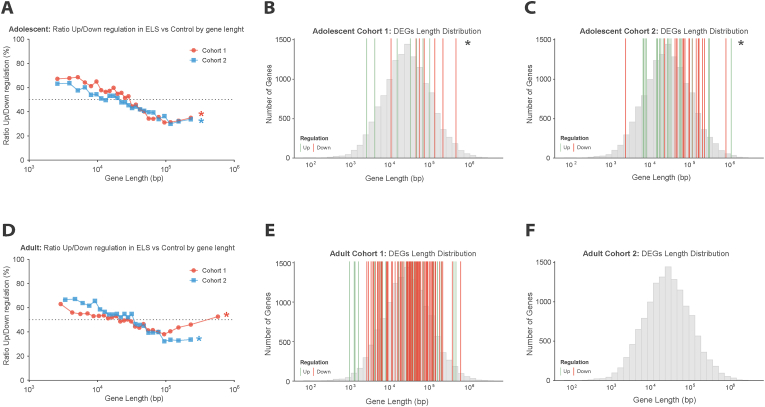
Early life stress induces a reproducible gene length-dependent transcriptional signature in the adolescent hippocampus. **A**. Ratio of upregulated to downregulated genes across gene length bins in ELS versus control animals for Adolescent cohort 1- (red) and Adolescent cohort 2 (blue). **B–C.** Gene length distribution of DEGs in Adolescent cohort 1 (B) and Adolescent cohort 2 (C). Gray histogram shows the background distribution of all genes. Vertical lines indicate individual DEGs colored by regulation direction: downregulated genes (red), upregulated genes (green). **D.** Ratio of upregulated to downregulated genes across the whole genome clustered by gene length bins in ELS versus control animals for Adult cohort 1 (São Paulo) (red) and Adult cohort 2 (Amsterdam) (blue). **E–F.** Gene length distribution of DEGs in Adult cohort 1 (São Paulo) (E) and Adult cohort 2 (Amsterdam)(F). Gray histogram shows the background distribution of all genes. Vertical lines indicate individual DEGs colored by regulation direction: downregulated genes (red), upregulated genes (green). Adolescent cohort 1: N_Control-Vehicle_ = 4 N_ELS-Vehicle_ = 4. Adolescent Cohort 2: N_Control-Vehicle_ = 6 N_ELS-Vehicle_ = 6. Adult cohort 1 (São Paulo): N_Control-Vehicle_ = 6 N_Control-RU486_ = 6 N_ELS-Vehicle_ = 6 N_ELS-RU486_ = 6. Adult cohort 2 (Amsterdam): N_Control-Vehicle_ = 6 N_Control-RU486_ = 6 N_ELS-Vehicle_ = 6 N_ELS-RU486_ = 6. **∗**: ELS effect. Effect p ≤ 0.05.

Therefore, we analyzed the relationship between gene length and differential expression in the two adolescent cohorts. We sorted all abundantly expressed genes (>1 transcript per million) in the hippocampus based on gene length and clustered them in bins of 500 genes (as developed by van Galen et al., 2025). This analysis revealed a gene length-dependent shift in the direction of transcriptional changes: short genes (<10 kb) were predominantly upregulated following ELS (ratio of upregulation: 60-70%), but this pattern reversed progressively with increasing gene length, with long genes (>100 kb) showing a preferential bias toward downregulation (30-40% upregulated versus downregulated; Fig. 4A). Both cohorts exhibited a significant gene length-dependent pattern (Adolescent cohort 1: r = −0.94, t(24) = −13.28, p < 0.001; Adolescent cohort 2: r = −0.93, t(24) = −12.29, p < 0.001), with marginally significant differences in slopes (F(1,48) = 8.46, p = 0.05).

This gene length-dependent signature was highly reproducible despite individual differences in specific DEGs between cohorts and was further supported by analysis of our functionally annotated genes, where downregulated genes trended toward or were significantly longer than upregulated genes in both cohorts (Adolescent cohort 1: W(7,7) = 11, p = 0.097, the non-significant p value likely due to limited sample size; Adolescent cohort 2: W(21,17) = 92, p = 0.010) (Fig. 4B–C). A mixed-effects model confirmed the consistency of this gene length effect across cohorts (β = −0.44, t(50) = −2.84, p = 0.007), with no significant difference between cohorts (β = 0.21, t(50) = 0.59, p = 0.555).

Finally, we examined whether this gene length-dependent signature extended into adulthood. In adult animals, both cohorts showed the same pattern of downregulation of long genes (Adult cohort 1 (São Paulo): r = −0.71, t(23) = −4.86, p < 0.001; Adult cohort 2 (Amsterdam): r = −0.97, t(23) = −17.82, p < 0.001). The substantial divergence in correlation strength was reflected in significantly different regression slopes (F(1,46) = 44.07, p < 0.001) (Fig. 4D).

Due to limited DEGs in the Adult cohort 2 (Amsterdam) (Fig. 4F), analysis was restricted to Adult Cohort 1 (São Paulo), which revealed no significant gene length bias (W(38,74) = 1312, p = 0.566) (Fig. 4E), indicating that the transcriptional constraint on (differential expressed) longer genes is specific to adolescent ELS.

This pattern is consistent with GLTD, which has been associated with accelerated biological aging (van Galen et al., 2025). To further explore this association, we performed GSEA using brain-derived cell type-specific aging signature gene sets from the Tabula Muris Senis dataset (The Tabula Muris Consortium, 2020). At PND31, microglia, astrocytes, and pericytes showed significant positive enrichment of aeing signatures in both cohorts (Supplementary Fig. 3), suggesting that ELS-induced transcriptional changes in the adolescent hippocampus recapitulate brain cell type-specific aging signatures. At PND120, this aging-like signature was largely attenuated, with no cell type reaching significance in both cohorts.

Overall, these findings suggest that ELS induces a reproducible gene length-dependent transcriptional signature in the hippocampus, characterized by preferential upregulation of shorter genes and progressive downregulation with increasing gene length, consistent with a gene-length-dependent transcription decline (GLTD) pattern. This transcriptional shift is accompanied by an enrichment of brain cell type-specific aging signatures, particularly in glial and vascular cell types during adolescence, linking ELS-induced GLTD to accelerated transcriptomic aging in the hippocampus.

## 4 Discussion

We set out to investigate how ELS long-lastingly affects the male mouse hippocampus at the transcriptome level, given the clear neuroendocrine and behavioral phenotype described already in the 1950s by Gig Levine and later by others (Levine, 1957, 2005; and e.g. reviewed for the LBN model in Walker et al., 2017). Transcriptome analyses of multiple cohorts confirmed that ELS/LBN can alter the hippocampal transcriptome, but (at least in adolescence) did not do so consistently. Thus, assessment of the hippocampal transcriptome in two separate yet highly comparable adolescent reproducibility cohorts showed that controlling external variables (lab environment, researcher, animal supplier, laterality, etc.) did not improve the consistency. Altogether, our data raise caution concerning the reproducibility and replicability, and thereby usability, of the current (commonly used) combination of ELS paradigm and molecular techniques to study molecular ELS effects in the hippocampus of male mice. Possibly, the inconsistencies are less of a concern when investigating the transcriptome at a more global level, because we did observe a reproducible transcriptional signature characterized by a shift in gene expression based on gene length after ELS.

### 4.1 Transcriptional effects of ELS and normalization by RU486

We opted to study the long-lasting transcriptional effects of ELS in male mice based on the reported behavioral effect after auditory fear conditioning and reversal thereof by GR antagonist RU486 intervention (Arp et al., 2016; Loi et al., 2017; Sanguino-Gómez and Krugers, 2024). The normalization of adult behavior by adolescent intervention indicates a mechanism of programming that involves the GR in this reversal phenomenon and therefore likely also in the initial effects of ELS. To study these long-lasting effects, we exposed the animals solely to ELS and adolescent treatment intervention, but not to other stressors or any behavioral task before sacrifice, which might alter the programming (further discussed below).

In adult mice, we observed long-lasting transcriptional effects of ELS on the dorsal hippocampus, yet these changes were not consistent across cohorts (116 vs. 0 DEGs, see discussion below).

While behavioral normalization by RU486 has been reported in several stress paradigms (Ding et al., 2019, 2020; Loi et al., 2017; Papilloud et al., 2019; Sanguino-Gómez and Krugers, 2024), though inconsistently (Kentrop et al., 2016; Sanguino-Gómez et al., 2024), our data show that RU486 does not restore ELS-induced transcriptomic alterations in bulk hippocampal homogenates at baseline. This is paradoxical: behavioral deficits can be ameliorated by RU486 (albeit inconsistently across studies), yet this does not translate to normalization of ELS-induced transcriptomic changes in the hippocampus. Notably, RU486 similarly fails to normalize transcriptional changes in the hippocampus after chronic stress (Datson et al., 2012).

This specific finding -the absence of molecular normalization at the transcriptomic level in this tissue at this timepoint-does not preclude several important possibilities: (1) normalization may occur in other brain regions critically involved in fear learning (Bhatnagar et al., 2000; Malter Cohen et al., 2013; Yang et al., 2015); (2) molecular normalization may be detectable at other regulatory levels (protein, epigenetic modifications, chromatin accessibility); (3) molecular normalization may be manifested only when animals face a behavioral challenge or acute stressor (as observed in the behavioral normalization of FC effects of RU486 across studies: Arp et al., 2016; Lesuis et al., 2019; Sanguino-Gómez and Krugers, 2024) or (4) normalization may be cell-type specific and obscured by bulk tissue analysis. As this work represents, to the extent of our knowledge, the first omics-level characterization of this ELS-RU486 intervention paradigm, comparable datasets are not available at the time of writing to explore these possibilities.

To distinguish between these possibilities, a more comprehensive and integrated approach is likely required, encompassing multiple levels of regulation, different brain areas, and cellular resolution. Such an approach could leverage multiple omics platforms per animal at single-cell resolution, enabling dissection of RU486's normalizing potential with greater depth, potentially requiring machine learning approaches to resolve the expected non-linear complexity of these systems.

### 4.2 Reproducibility of transcriptional changes after ELS

A remarkable finding of this study is the lack of reproducibility in genome-wide transcriptional changes after ELS. While Cohort 1 (São Paulo) identified 116 DEGs, Cohort 2 (Amsterdam) showed no significant differential gene expression. Only 60 genes (51.7%) had consistent directionality between cohorts -a proportion indistinguishable from chance - and these genes showed, in Cohort 2, non-significant, modest effects (FDR ≥0.99; mean |log2FC| = 0.066). This inconsistency occurred despite both cohorts being processed by the same experimenter, although differing in laboratory location and hippocampal hemisphere analyzed.

In the two adult cohorts, tissue was collected in different labs (The Netherlands and Brazil), and while the key stress-inducing components of the LBN paradigm were strictly followed for all cohorts and experiments were performed by the same investigator (EHLU), several factors may have contributed to this variability.

Firstly, we compared data acquired from the right (Cohort 1; São Paulo) versus the left (Cohort 2; Amsterdam) dorsal hippocampus. Although hemispheric differences in hippocampal transcriptomics have not been systematically characterized following stress, hemispheric differences in hippocampal volume and activity-dependent c-fos expression have been described following early stress, with the strongest effects observed in the right hemisphere (Loi et al., 2017), consistent with findings after chronic stress in the prefrontal cortex (Czéh et al., 2007). In the transcriptome, it is known that early postnatal gene expression differs substantially between left and right hippocampus (Moskal et al., 2006) and that the right amygdala shows enhanced vulnerability to early stress (Guadagno et al., 2020). Cohort 1 (São Paulo; right hippocampus) showed substantially more DEGs than Cohort 2, a pattern consistent with greater right-hemispheric sensitivity to stress. Secondly, the experiments were conducted in two different locations (The Netherlands and Brazil), with differences in climate and seasonality that could introduce variations in stress exposure. However, animal facilities in both locations maintained standardized environmental conditions and every precaution was taken to minimize extraneous stressors, and no significant incidents were registered in either facility. Overall, one could reason that if ELS-induced transcriptional changes are sufficiently robust to drive the behavioral consequences we and others have documented, they should survive such variations in experimental details.

Nevertheless, to circumvent these and other potential sources of variation, two parallel experiments were carried out in the same lab, by the same experimenter, the same animal supplier, and on the same hemisphere (right). We also examined genome-wide transcriptional effects earlier on, arguing that some of the changes may be less resistant to the process of maturation and aging. And despite these additional standardization steps, transcriptional changes in the hippocampus after ELS remained largely inconsistent. While these adolescent cohorts showed improved directionality consistency (66.7% vs. 51.7% in adults) and larger effect sizes (mean |log2FC| = 0.21 vs. 0.066), only 2 genes (*Lct,* downregulated and *Meg3,* upregulated) achieved statistical significance in both cohorts, with the remaining consistent genes showing p-values far from significance and modest fold changes.

Interestingly, lactase (*Lct*) downregulation was consistently observed in both adolescent cohorts following ELS. This finding is particularly intriguing given that, within the brain, lactase is only expressed in the dorsal hippocampus (Fanselow and Dong, 2010; Jaszczyk et al., 2022; Thompson et al., 2020), where its function remains unknown, although it has been hypothesized to serve as a signaling molecule (Thompson et al., 2020). In the intestine, lactase undergoes developmentally programmed downregulation around the weaning period (Troelsen et al., 2003; Kuokkanen et al., 2006). The observation of enhanced hippocampal lactase downregulation at PND31 in ELS animals may reflect accelerated maturation induced by early-life adversity (consistent with Bath et al., 2016), suggesting that stress-induced transcriptional changes might amplify (and accelerate) normal developmental programs active during this critical transition period.

Beyond this two-genes exception, the limited cross-cohort reproducibility of individual genes warrants consideration of several potential explanations for the overall lack of reproducibility. Firstly, we cannot rule out that the relatively limited cohort sizes play a role, a well-documented problem in animal research, including studies on ELS (Bonapersona et al., 2021). Of note, our current group sizes (between 4 and 6 animals per group) were relatively large compared to other studies using similar technologies. Interestingly, pooling both adolescent cohorts identified over two-fold the number of DEGs. However, pooling also resulted in the loss of significance for transcripts that were identified as such in the separate per-cohort analyses. These might entail false positives, which were identified due to the increased power, but they can also indicate that the transcriptome effects of ELS can differ per cohort. Secondly, potential effects in a subset of cells may have become diluted in our hippocampus-based approach. Possibly, transcriptome analyses in (a limited set of homogeneous) single cells may lead to more robust effects. Thirdly, there may be factors–unbeknownst to us– that we did not control. Finally, we cannot exclude that ELS might influence the expression of two sets of genes: On the one hand, a tightly regulated set of genes (e.g. small effect sizes) underlying the core ELS effects; and, on the other hand, a set of genes that stochastically differ per cohort. It is conceivable that exposure of animals to an acute stressor (e.g. footshock in the fear conditioning experiments – Arp et al., 2016; Sanguino-Gómez et al., 2024) reduces the degree of stochasticity, thus yielding more robust and reproducible findings, as was e.g. shown after chronic stress (Datson et al., 2013).

### 4.3 Gene length-dependent transcriptional signature following ELS

Despite limited overlap in individual DEGs between cohorts, we identified a consistent gene-length-dependent transcriptional pattern following ELS in both adolescent cohorts, with a similar observation in the adult data. This reproducible signature was characterized by preferential upregulation of short genes and progressive downregulation of longer genes, consistent with gene-length-dependent transcriptional decline (GLTD). Critically, this pattern emerged despite the presumed stochasticity of individual gene responses and survives despite substantial methodological differences between cohorts (researcher, laboratory location, hippocampal hemisphere analyzed and developmental stage), revealing a possible underlying systematic vulnerability based on gene length caused by ELS.

This vulnerability may result from multiple, potentially complementary mechanisms. First, metabolic and energetic constraints due to early-life adversity (Salmina et al., 2021) could play a primary role. As transcribing long genes is energetically costly and time-consuming (Wagner, 2005), upon ELS, cells may prioritize transcription of shorter genes necessary for immediate survival responses at the expense of longer, more transcriptionally demanding genes. This is particularly interesting given that many (stress-responsive) immediate early genes (IEGs) are characteristically short (<5 kb) and enable rapid transcriptional responses to novel stimuli or stress (Lopes et al., 2021). In fact, in the adolescent cohorts, several of these genes are consistently upregulated, including *Fos* (3.4 kb), *Npas4* (5.9 kb) and *Arc* (3.5 kb), reflecting a transcriptional prioritization of immediate stress-responses. Notably, this elevated IEG expression was observed at baseline in the absence of acute stress exposure, suggesting that ELS might induce a persistent state of heightened threat responsiveness in the adolescent hippocampus even in safe environments (consistent with Arp et al., 2016).

As a consequence, the sustained energetic burden of maintaining elevated short genes (IEG, among others) transcription might divert finite cellular resources away from longer genes, which, from an evolutionary and functional perspective, are highly enriched for specific functions critical to neural development and plasticity. Neurons are particularly enriched in the expression of long genes, with neuronal genes being significantly longer on average than those expressed in other cell types (Gabel et al., 2015; Lopes et al., 2021; Zylka et al., 2015). Long genes disproportionately encode synaptic proteins, cell adhesion molecules, axon guidance factors, and transcription factors essential for neurodevelopment (Gabel et al., 2015; Soheili-Nezhad et al., 2024; King et al., 2013). These genes are often under tight regulatory control and show precise spatiotemporal expression patterns during brain development. However, upon ELS, resource allocation constraints may shift transcription from genes supporting growth and complex neural development toward those enabling immediate maintenance and survival.

Alternatively, the preferential downregulation of longer genes may reflect their increased susceptibility to transcription-blocking damage. Longer genes accumulate more stochastic lesions during cellular stress and require substantially more time for transcriptional completion, making them particularly vulnerable to stress-induced disruptions (Vermeij et al., 2016; Soheili-Nezhad et al., 2024). The resulting preferential downregulation of long genes could therefore have far-reaching consequences for neural circuit formation, synaptic function, and ultimately behavior; effects that may manifest across the lifespan. Moreover, biological pathways that involve multiple sequential steps may be particularly vulnerable to ELS-induced transcriptional changes, as disruption of any component gene could compromise the entire pathway's function.

From a methodological perspective, we must also consider analytical effects inherent to RNA-seq. Given the relative nature of RNA-seq data, the apparent magnitude of the gene length-dependent pattern we observe may be influenced by normalization procedures. When short genes are preferentially upregulated, longer genes consequently appear relatively downregulated during normalization even if their absolute expression levels do not decline proportionally, and vice versa. However, this does not negate the underlying biological phenomenon; rather, it suggests that the true gene length-dependent constraint may be somewhat amplified or rendered more apparent through the relative scaling effects intrinsic to RNA-seq analysis.

Importantly, GLTD has been mechanistically linked to biological aging (Vermeij et al., 2016; Gyenis et al., 2023; van Galen et al., 2025). This is particularly relevant given that ELS has been associated with accelerated hippocampal maturation (Bath et al., 2016) and aging-associated changes such as progressive hippocampal dendritic atrophy (Brunson et al., 2005). In humans, childhood adversity has been linked to accelerated biological aging, including telomere erosion (Ridout et al., 2018) and epigenetic age acceleration as measured by the Horvath DNA methylation clock (Jovanovic et al., 2017). Our GSEA results extend these findings to the transcriptomic level, showing that a one-week postnatal stress exposure is sufficient to produce an aging-like transcriptomic fingerprint in the adolescent hippocampus, particularly in glial and vascular cell types such as microglia, astrocytes, and pericytes. The attenuation of this signature in adulthood suggests that ELS-induced transcriptomic aging may represent an early vulnerability window rather than a permanent state, although it remains to be determined whether this apparent recovery reflects true normalization or compensatory transcriptional mechanisms.

Overall, the consistency of this gene length-dependent pattern across cohorts, despite the inconsistency of individual gene changes, suggests that this systematic bias may represent a more stable molecular signature of ELS than individual gene expression alterations. Rather than programming specific genes, ELS may impose a constraint on transcriptional capacity that disproportionately affects longer, more transcriptionally demanding genes. For instance, lactase (*Lct*), which showed consistent significant downregulation in both adolescent cohorts, is a long gene (∼49 kb). Importantly, this may reflect a global transcriptional constraint, not selective pathway bias, with functional selectivity emerging from the genomic architecture of long and short genes rather than from specific, targeted transcriptional regulation. This interpretation reconciles the apparent contradiction between robust behavioral effects of ELS reported in the literature and the variable (stochastic) molecular signatures we observed: the functional consequences may arise not from consistent changes in specific genes, but from a consistent bias affecting a functionally important set of genes defined by their length.

Future studies should investigate whether this gene length-dependent vulnerability occurs in other brain regions affected by ELS, and whether the gene length bias is particularly pronounced in specific neuronal populations or distributed across multiple cell types in the hippocampus. Additionally, it remains to be determined whether interventions like RU486, which have proven efficient for associated behavioral consequences of early-life adversity in some studies (Arp et al., 2016; Loi et al., 2017; Sanguino-Gómez and Krugers, 2024) can prevent or reverse this molecular bias.

## 5 Conclusion

Altogether, we conclude that variation is inherent to the current combination of ELS and transcriptional investigation, especially when examining a single (and relatively small) cohort. This should be taken into account when designing future studies, and emphasizes the importance of including a substantial number of animals per experimental group, even when the experiments are costly and a high number of genes per animal are investigated. Although a certain degree of variability of ELS effects - whether it concerns behavior, corticosterone levels, or gene expression - is well described and has previously been critically assessed (Murthy and Gould, 2018; Walker et al., 2017), a more comprehensive and integrated approach might be necessary if we want to better understand the molecular mechanism underlying the long-lasting effects of ELS and the normalization effects of a brief RU486 treatment during the adolescence period.

From a translational perspective, the high degree of variability in specific genes is interesting. Not every individual who experiences adversity in early life will develop behavioral and/or emotional deficits later on, although, at a group level, early-life adversity is a well-established risk factor for psychiatric disorders (Kessler et al., 2010; LeMoult et al., 2020; Teicher et al., 2016). We propose that a potential element of stochasticity can, therefore, not be entirely ruled out in humans, too.

However, despite this individual gene-level stochasticity, our identification of a consistent gene-length-dependent transcriptional decline (GLTD) provides a mechanism to explain the robust behavioral effects of ELS. The preferential downregulation of longer genes (and upregulation of short ones) represents a systematic constraint that could drive significant functional consequences without requiring consistent changes in specific genes.

## CRediT authorship contribution statement

**Jeniffer Sanguino-Gómez:** Conceptualization, Data curation, Formal analysis, Investigation, Project administration, Validation, Visualization, Writing – review & editing. **Jacobus C. Buurstede:** Data curation, Formal analysis, Investigation, Methodology, Visualization, Writing – original draft, Writing – review & editing. **Eduardo H.L. Umeoka:** Conceptualization, Investigation, Writing – review & editing. **Marcia Santos da Silva Umeoka:** Conceptualization, Investigation, Writing – review & editing. **Max Gentenaar:** Formal analysis. **Norberto Garcia-Cairasco:** Writing – review & editing. **Mario F. Juruena:** Writing – review & editing. **Wilbert P. Vermeij:** Conceptualization, Formal analysis, Methodology, Writing – review & editing. **Jan H.J. Hoeijmakers:** Conceptualization, Writing – review & editing. **Harm J. Krugers:** Conceptualization, Writing – review & editing. **Marian Joëls:** Conceptualization, Funding acquisition, Project administration, Supervision, Validation, Writing – original draft, Writing – review & editing. **Onno C. Meijer:** Conceptualization, Funding acquisition, Methodology, Project administration, Resources, Supervision, Validation, Writing – original draft, Writing – review & editing.

## Funding

J.S.G. was supported by the Fundación Mutua Madrileña (grant BP173352019) and Corcept Therapeutics. M.J. was supported by the Consortium on Individual Development (CID), funded through the Gravitation program of the Dutch Ministry of Education, Culture, and Science and the Netherlands Organization for Scientific Research (NWO; grant 024.001.003). E.U. was supported by the São Paulo Research Foundation (Fundação de Amparo à Pesquisa do Estado de São Paulo – FAPESP; grants 2015/18773-1 and 2016/21369-0).

## Declaration of competing interest

O.C.M receives funding from Corcept Therapeutics, USA, which develops glucocorticoid receptor modulators for clinical use. The other authors declare no competing interests.

## Data Availability

Data will be made available on request.

## Appendix A Supplementary data

Multimedia component 5 Multimedia component 5

Fig. S1 Principal component analysis of hippocampal transcriptomes across cohorts and developmental stages. A. PCA of Adult cohort 1 (São Paulo) showing distribution of samples along PC1 (44% variance) and PC2 (11% variance). **B.** PCA of Adult cohort 2 (Amsterdam) showing distribution of samples along PC1 (47.61% variance) and PC2 (20.2% variance). **C.** PCA of Adolescent cohort 1 showing distribution of samples along PC1 (37.25% variance) and PC2 (8.22% variance). **D.** PCA of Adolescent cohort 2 showing distribution of samples along PC1 (35.32% variance) and PC2 (18.67% variance). dolescent cohort 1: N_Control-Vehicle_ = 4 N_ELS-Vehicle_ = 4 Adolescent cohort 2: N_Control-Vehicle_ = 6 N_ELS-Vehicle_ = 6 Adult cohort 1 (São Paulo): N_Control-Vehicle_ = 6 N_Control-RU486_ = 6 N_ELS-Vehicle_ = 6 N_ELS-RU486_ = 6 Adult cohort 2 (Amsterdam): N_Control-Vehicle_ = 6 N_Control-RU486_ = 6 N_ELS-Vehicle_ = 6 N_ELS-RU486_ = 6. Fig. S1

Fig. S2 Early life stress cohorts sacrificed at PND31 have the same body weight loss as the ones sacrificed at PND120. A-B. Body weights (in grams ± SEM) of experimental animals measured at PND 2 (prior to group assignment) and PND 9 (immediately following control or ELS rearing conditions) and adolescence (average PND 28-30, when treatment was administered) in Adolescence cohort 1 (A) and in an independent validation cohort Adolescence cohort 2 (B). Adolescent cohort 1: N_Control-Vehicle_ = 4 N_ELS-Vehicle_ = 4 Adolescent cohort 2: N_Control-Vehicle_ = 6 N_ELS-Vehicle_ = 6 **∗**: ELS effect. Effect p ≤ 0.05. Fig. S2

Fig. S3 **Gene-set enrichment analysis of brain cell type-specific aging signatures across adolescent and adult ELS cohorts.** Normalized enrichment scores (NES) for aging gene sets derived from brain cell types in the Tabula Muris Senis dataset. GSEA was performed on ELS vs Control (Vehicle-only) comparisons for each cohort. Asterisks indicate Benjamini-Hochberg adjusted p-values (∗p < 0.05, ∗∗p < 0.01, ∗∗∗p < 0.001). Positive NES (red) indicates enrichment of aging-associated genes among genes upregulated in ELS; negative NES (blue) indicates enrichment among downregulated genes Adolescent cohort 1: N_Control-Vehicle_ = 4 N_ELS-Vehicle_ = 4 Adolescent cohort 2: N_Control-Vehicle_ = 6 N_ELS-Vehicle_ = 6 Adult cohort 1 (São Paulo): N_Control-Vehicle_ = 6 N_Control-RU486_ = 6 N_ELS-Vehicle_ = 6 N_ELS-RU486_ = 6 Adult cohort 2 (Amsterdam): N_Control-Vehicle_ = 6 N_Control-RU486_ = 6 N_ELS-Vehicle_ = 6 N_ELS-RU486_ = 6. Fig. S3
