# Supplementary figures and images for: Consistent changes in global gene expression patterns despite strong variation in individual gene expression in the male mouse hippocampus following early life stress
Source: Neurobiol Stress. 2026 Mar 30;42:100808. doi: 10.1016/j.ynstr.2026.100808 (PMC13085001; doi:10.1016/j.ynstr.2026.100808)

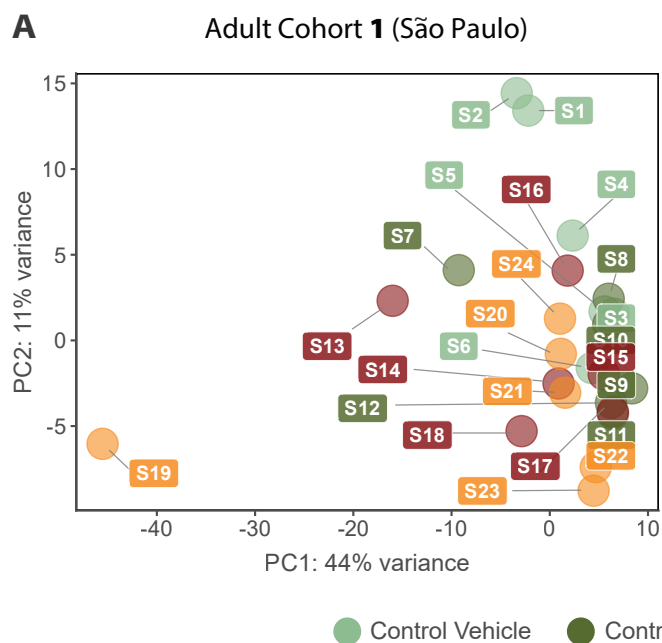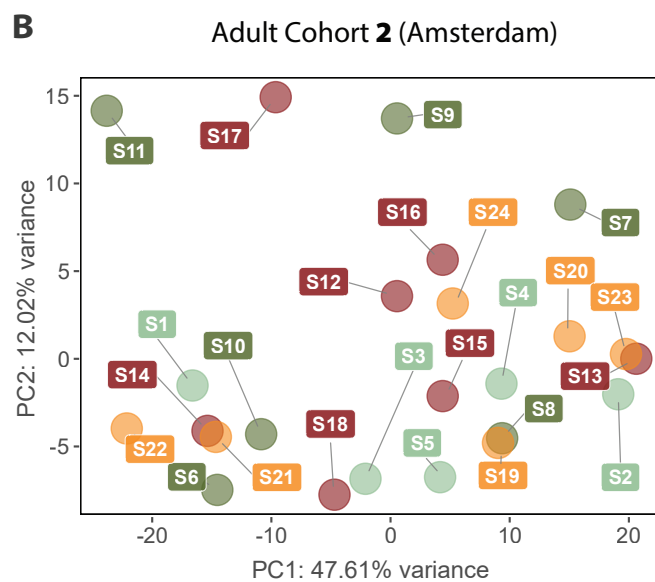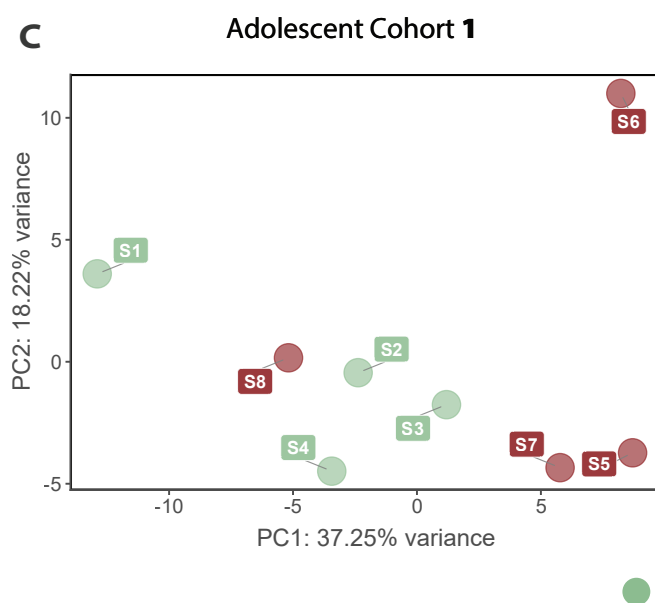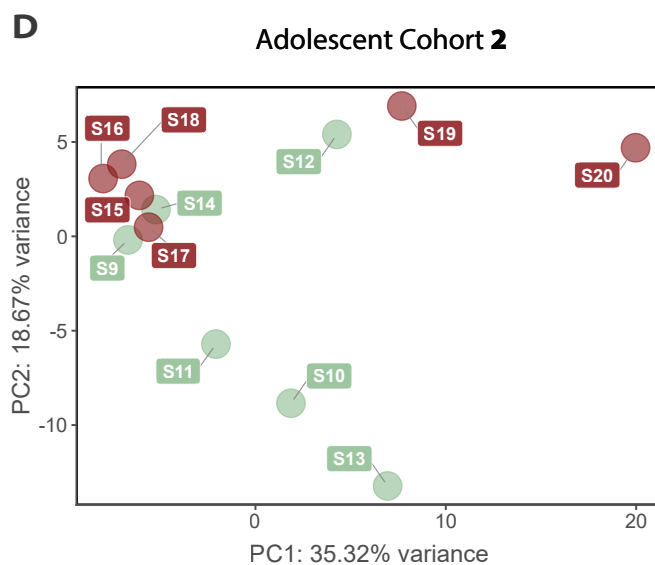

Supplement: Fig. S1 — Principal component analysis of hippocampal transcriptomes across cohorts and developmental stages. A. PCA of Adult cohort 1 (São Paulo) showing distribution of samples along PC1 (44% variance) and PC2 (11% variance). B. PCA of Adult cohort 2 (Amsterdam) showing distribution of samples along PC1 (47.61% variance) and PC2 (20.2% variance). C. PCA of Adolescent cohort 1 showing distribution of samples along PC1 (37.25% variance) and PC2 (8.22% variance). D. PCA of Adolescent cohort 2 showing distribution of samples along PC1 (35.32% variance) and PC2 (18.67% variance). dolescent cohort 1: NControl-Vehicle = 4 NELS-Vehicle = 4 Adolescent cohort 2: NControl-Vehicle = 6 NELS-Vehicle = 6 Adult cohort 1 (São Paulo): NControl-Vehicle = 6 NControl-RU486 = 6 NELS-Vehicle = 6 NELS-RU486 = 6 Adult cohort 2 (Amsterdam): NControl-Vehicle = 6 NControl-RU486 = 6 NELS-Vehicle = 6 NELS-RU486 = 6. [file mmc6.pdf]

TMS Brain Cell-Type Aging Signatures  
(ELS vs Control)

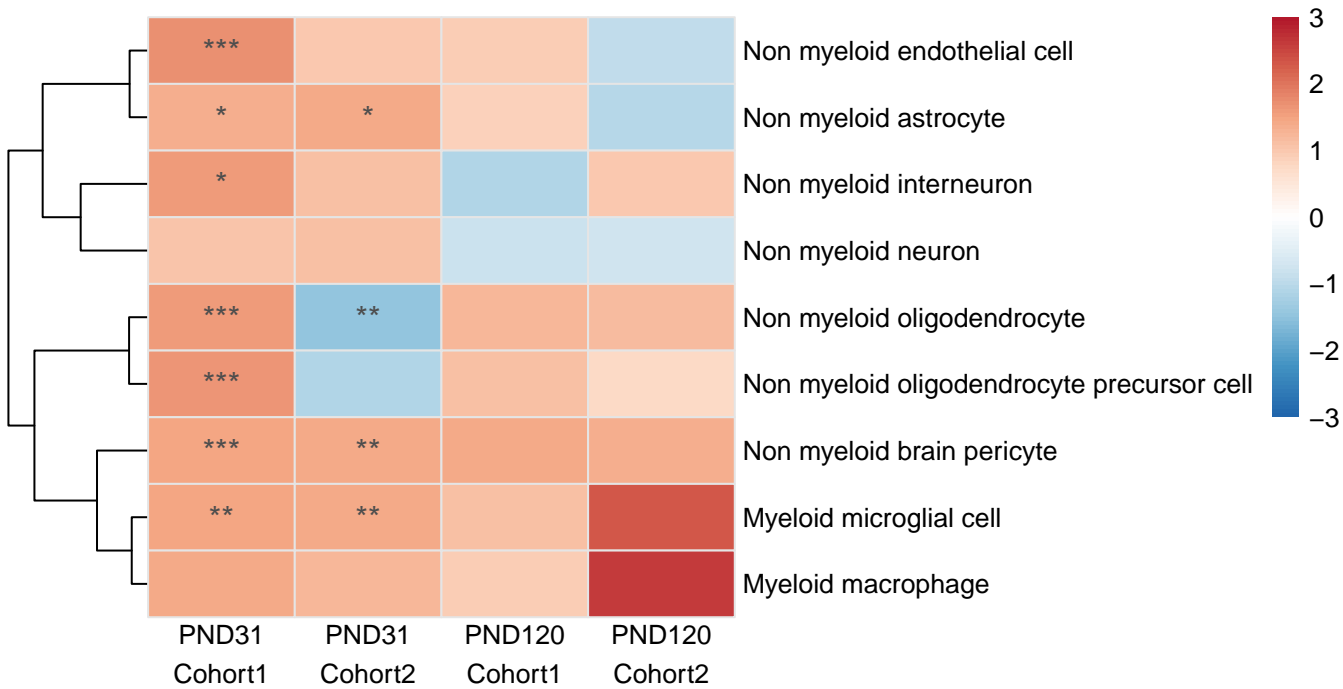

Supplement: Fig. S3 — Gene-set enrichment analysis of brain cell type-specific aging signatures across adolescent and adult ELS cohorts. Normalized enrichment scores (NES) for aging gene sets derived from brain cell types in the Tabula Muris Senis dataset. GSEA was performed on ELS vs Control (Vehicle-only) comparisons for each cohort. Asterisks indicate Benjamini-Hochberg adjusted p-values (∗p < 0.05, ∗∗p < 0.01, ∗∗∗p < 0.001). Positive NES (red) indicates enrichment of aging-associated genes among genes upregulated in ELS; negative NES (blue) indicates enrichment among downregulated genes Adolescent cohort 1: NControl-Vehicle = 4 NELS-Vehicle = 4 Adolescent cohort 2: NControl-Vehicle = 6 NELS-Vehicle = 6 Adult cohort 1 (São Paulo): NControl-Vehicle = 6 NControl-RU486 = 6 NELS-Vehicle = 6 NELS-RU486 = 6 Adult cohort 2 (Amsterdam): NControl-Vehicle = 6 NControl-RU486 = 6 NELS-Vehicle = 6 NELS-RU486 = 6. [file mmc8.pdf]
