# Supplementary material for: Consistent changes in global gene expression patterns despite strong variation in individual gene expression in the male mouse hippocampus following early life stress
Source: Neurobiol Stress. 2026 Mar 30;42:100808. doi: 10.1016/j.ynstr.2026.100808 (PMC13085001; doi:10.1016/j.ynstr.2026.100808)
Supplement: Fig. S2 — Early life stress cohorts sacrificed at PND31 have the same body weight loss as the ones sacrificed at PND120. A-B. Body weights (in grams ± SEM) of experimental animals measured at PND 2 (prior to group assignment) and PND 9 (immediately following control or ELS rearing conditions) and adolescence (average PND 28-30, when treatment was administered) in Adolescence cohort 1 (A) and in an independent validation cohort Adolescence cohort 2 (B). Adolescent cohort 1: NControl-Vehicle = 4 NELS-Vehicle = 4 Adolescent cohort 2: NControl-Vehicle = 6 NELS-Vehicle = 6 ∗: ELS effect. Effect p ≤ 0.05. [file mmc7.pdf]

A

Adolescent Cohort 1						
Period	PND 2		PND 9		PND 28	
Group	Control	ELS	Control	ELS	Control	ELS
Body weight (g)	1.49 ± 0.06	1.32 ± 0.04	5.01 ± 0.17	3.26 ± 0.17 (*)	15.70 ± 0.35	12.85 ± 0.56 (*)

B

Adolescent Cohort 2						
Period	PND 2		PND 9		PND 28	
Group	Control	ELS	Control	ELS	Control	ELS
Body weight (g)	1.41 ± 0.02	1.40 ± 0.02	4.51 ± 0.19	3.06 ± 0.22 (*)	15.23 ± 0.34	13.57 ± 0.57 (*)
